# P-1840. Differential Memory CD4 T Cell Responses to SARS-CoV-2 in Children and Adults Pre- and Post-Pandemic

**DOI:** 10.1093/ofid/ofae631.2003

**Published:** 2025-01-29

**Authors:** Regina K Rowe, Nelson Huertas, Bailey Matthews, Jennifer L Nayak

**Affiliations:** University of Rochester Medical Center, Rochester, New York; University of Rochester Medical Center, Rochester, New York; University of Rochester Medical Center, Rochester, New York; University of Rochester Medical Center, Rochester, New York

## Abstract

**Background:**

Coronaviruses are endemic in the human population and include both seasonal viruses as well as the SARS-CoV-2 virus responsible for the COVID-19 pandemic. As a betacoronavirus, SARS-CoV-2 is closely related to two endemic coronaviruses (OC43 and HKU1), with exposure history differing between children and adults. We sought to identify age-specific differences in memory CD4 T cells specific for the SARS-CoV-2 Spike protein (S), as quantitative and qualitative differences in immune function may be responsible for age-related variation in disease presentation.

**Figure:**
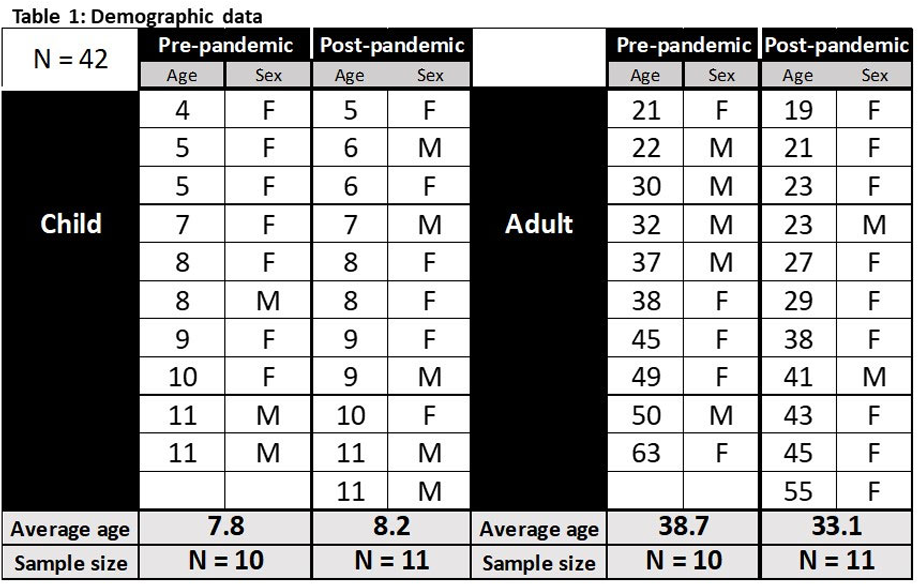

**Methods:**

Peripheral blood mononuclear cells were obtained from cohorts of children and adults enrolled pre-2019 and following the SARS-CoV-2 pandemic. Subjects enrolled post-pandemic had a history of COVID-19 vaccination. Cells were stimulated with peptide pools representing the unique and more cross-reactive portions of the S protein for 24 hours, with cytokine release blocked for the last 4 hours of coculture. Subsets of S-specific CD4 T cells were identified using a 31-plex spectral flow cytometry panel.

**Figure:**
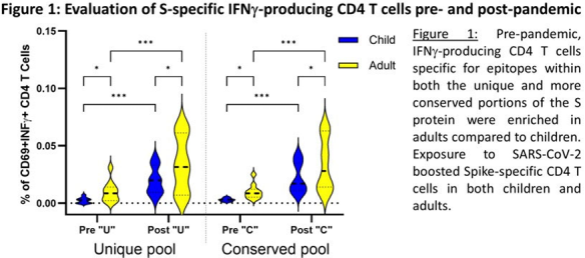

**Results:**

Pre-pandemic, adults had a higher frequency of activated IFNγ+ CD4 T cells specific for the S protein compared to children, with boosting of responses in both adults and children following SARS-CoV-2 exposure. Overall, the CD4 T cell response to the S protein was Th1 biased, with adults having a greater proportion of cells expressing IFNγ, IL2 and TNFα. When cytokine-expressing, antigen-specific CD4 T cells were subsetted by CD45RA and CCR7 expression, there was a more naïve phenotype pre-pandemic, with transition to an effector memory phenotype post-2019.

**Figure:**
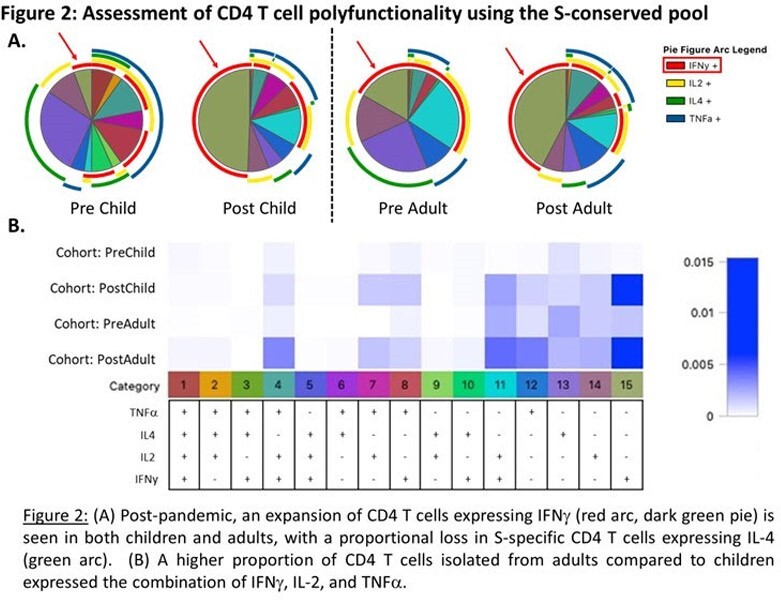

**Conclusion:**

A greater magnitude of CD4 T cells specific for the S protein was detected in adults pre-pandemic, with this reactivity boosted in both children and adults following SARS-CoV-2 exposure. Boosting of reactivity to both unique and conserved S protein epitopes in children and adults suggested that exposure to SARS-CoV-2 via vaccination did not preferentially boost memory CD4 T cells specific for conserved epitopes. Post-pandemic, there was a shift to a more Th1 phenotype together with a gain in cytokine-producing CD4 T cells with a phenotype consistent with effector memory.

**Figure:**
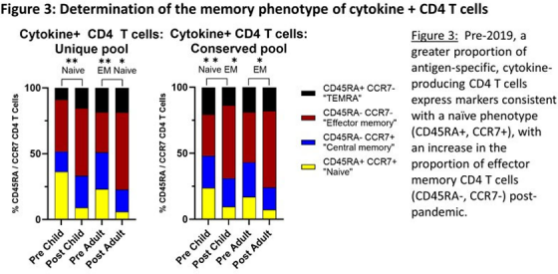

**Disclosures:**

**Jennifer L. Nayak, MD**, Merck, Inc.: Grant/Research Support|Moderna, Inc.: Grant/Research Support|Pfizer, Inc.: Grant/Research Support

